# Safety of Oral Food Challenges in Early Life

**DOI:** 10.3390/children5060065

**Published:** 2018-05-30

**Authors:** Katherine Anagnostou

**Affiliations:** 1 Section of Immunology, Allergy and Rheumatology, Department of Pediatrics, Texas Children’s Hospital, Houston, TX 77030, USA; Aikaterini.Anagnostou@bcm.edu; Tel.: +1-832-824-1319; 2 ection of Immunology, Allergy and Rheumatology, Department of Pediatrics, Baylor College of Medicine, Houston, TX 77030, USA

**Keywords:** oral food challenge, food allergy, infant, anaphylaxis

## Abstract

Oral food challenges are becoming more frequent in the allergy clinic due to an increased demand related to early food introduction in infants. We examined the safety of oral food challenges in 18 high-risk infants with prior allergic reactions, as well as infants with no known exposure to the food, presenting consecutively in a dedicated food allergy clinic for an oral food challenge. Foods challenged included peanut, tree nuts, sesame, baked egg, baked milk, and soy. A total of 17/18 (94%) infants had a negative challenge. Only 1/18 (6%) had a positive challenge, and in this case, symptoms were mild and limited to the skin. Our results suggest that food challenges in infants and young children up to the age of 2 years are safe with symptoms limited to the skin when reactions occur. In our cohort, the large majority of food challenges were negative, with most infants being sensitized rather than allergic to the food. Larger studies are needed to confirm this finding.

## 1. Introduction

Food allergies are common and increasing in prevalence, representing a major health concern in many countries around the world. In the United States, approximately 4–8% of children are affected, with the prevalence of food allergy being highest in infants and toddlers, with the ‘8 major allergenic foods’ in childhood being milk, egg, peanut, tree nuts, shellfish, fish, wheat, and soy [1]. Food allergies pose a significant burden on the affected patients and their families [2]. Dietary restrictions, high levels of anxiety, and social limitations are common problems resulting in decreased quality of life. It is therefore of crucial importance to make the correct diagnosis and, currently, the gold standard test for food allergy diagnosis is an ‘oral provocation challenge’ or ‘food challenge’. An oral food challenge (OFC) is the process of administering gradually increasing doses of a food allergen up to a full age-appropriate portion, under medical supervision. Patients are observed for any signs or symptoms of an allergic reaction, which would be interpreted as a ‘positive’ challenge (‘allergic patient’). If no signs or symptoms occur during dose administration and for 2 h after ingesting a full portion of the food, the challenge is considered ‘negative’ and the patient not allergic [3].

Oral food challenges in infants and young children are becoming an increasingly common occurrence in the allergy clinic. This is the result of various factors including (a) recent significant changes in our approach to allergenic food introduction with the aim to prevent the development of food allergy; (b) reported important advantages in baked milk and egg introduction into the diet of milk and egg allergic children; and (c) importance of differentiating between sensitization (positive skin tests or specific IgE to the food without clinical reactivity) and true allergy (clinical reactivity to the food) in early life.

## 2. Methods

Our study examined the safety of infant food challenges. The study was approved by the Institutional Research Board committee at Baylor College of Medicine (protocol H-40400).We have established an in-hospital dedicated pediatric clinic, available since May 2017, to encourage early allergenic food introduction in high-risk infants that would be able to tolerate this. Infants are defined as ‘high-risk’ if they suffer from moderate/severe or early onset eczema, an existing food allergy, or both. The clinic also supports introduction of baked milk and egg to infants with the aim to liberate the diet and accelerate tolerance. Food challenges are also performed in order to differentiate between sensitization and true allergy in very young age. 

We collected data prospectively on the first 18 patients, up to the age of 2 years, who underwent food challenges, with the objective to evaluate safety of this procedure in this age group. All food challenges were performed in accordance with the PRACTALL guidelines [3]. Materials used for food challenges included smooth peanut or tree nut butter (diluted in milk, juice, or smoothie), tree nut milk (i.e., almond milk), cupcake containing milk or egg (for the baked challenges), soy milk, and tahini (for sesame). Skin prick testing to both the food extract and the fresh food were performed on the day of the challenge. For baked challenges, skin prick testing also included the baked form of the food (milk or egg). All necessary safety measures were in place prior to conducting the challenges. A dedicated clinic area was used, within the existing allergy clinic for challenges. Resuscitation facilities were available in case of severe allergic reactions, as well as access to a fully staffed Pediatric Intensive Care Unit (PICU) in case of severe anaphylaxis. (on a different floor of the same hospital where the clinic is located). Both physician and nursing cover was available for the challenges. (ratio: 1 physician and 1 nurse per 3–4 patients).

## 3. Results

We prospectively collected data on the first 18 children, up to the age of 2 years, who underwent an open, graded, oral food challenge (OFC) in our clinic for this study (see Table 1). 

In this cohort, median age was 13 months, range: 8 months–2 years. Male to female ratio was 8:1.

With regards to atopic history, the overwhelming majority of our patients (16/18, 89%) had eczema, but only 2 had a history of wheeze and only 1 a history of allergic rhinitis. A total of 14/18 (78%) reported another food allergy (different from the challenge food). Diagnosis of all atopic conditions were made and documented previously by an allergist.

Foods challenges included peanut in 6 children (33%), baked milk in 3 (17%), soy in 2 (11%), almond in 2 (11%), baked egg in 1 (5%), hazelnut in 1 (5%), walnut in 1 (5%), cashew in 1 (5%), and sesame in 1 (5%). 

Median skin prick test (SPT) values and ranges were: 3 mm for the food extract (range 0–20 mm), 0 mm for the fresh food (range 0–18 mm), and 7 mm for the baked form of the food tested (range 0–9 mm). Specific IgE values were available for 16/18 infants prior to the challenge. Median value was 0.34 kU/L (range 0.1–11.1 kU/L).

A total of 17/18 (94%) infants had a negative challenge. Only 1/18 (6%) had a positive challenge and in this case, symptoms were mild and limited to the skin (erythema and hives). Symptoms occurred within 1 hour and were treated successfully with a single dose of oral antihistamine (Benadryl). There were no biphasic reactions. 

In 11/18 (61%), the food had not been ingested previously. Patients with a prior history of allergic reaction reported the following symptoms: urticaria (5/18), vomiting (1/18), eczema flare (2/18), difficulty breathing (2/18), angioedema (1/18), itching (1/18), irritability (1/18). The patient who had a positive challenge reported hives only with his initial reaction. Both infants with reported difficulty in breathing as part of their initial reaction had negative challenges to the food.

## 4. Discussion

There have been significant changes recently in the recommendations for infant weaning, with early allergenic food introduction strongly encouraged, especially with regards to peanut, contrary to previous recommendations. A consensus document [4] published in January 2017 advises peanut introduction into the infant diet between the ages of 4 and 11 months. This recommendation is particularly relevant for high-risk infants, defined as those with severe eczema, egg allergy, or both. This recent change in practice, also supported by the American Academy of Pediatrics, was based on the results of the Learning Early About Peanut (LEAP) clinical trial [5] which showed that early introduction of peanut in high-risk infants resulted in significantly reduced risk of peanut allergy development by age 5 years.

Following publication of the new guidelines for peanut introduction, we anticipated an increasing number of peanut challenges in infants and young children, especially those belonging to the ‘high-risk’ category, and set up an early introduction clinic to meet this demand. Peanut was the most common food challenged in our clinic cohort. All our patients had negative peanut challenges. Most (83%) had never ingested peanut previously, but all had moderate–severe eczema and 3 had another food allergy (egg, milk, and tree nut). Skin tests to peanut extract revealed results of 0–4 mm, but interestingly, we noted mostly (67%) negative skin tests to the fresh form of peanut (usually peanut butter). The same observation was made for most of the tree nuts challenged (hazelnut, almond, and walnut). This may reflect a better predictive value for the fresh form of nut (compared to the extract), but larger patient groups will be required to further investigate this finding.

In addition to peanut, we opted to include baked milk and egg challenges, as research suggests that up to 70% of milk- and egg-allergic children are able to tolerate the baked form of these foods [6]. This allows for a more liberated diet, nutritional benefits, less social restrictions, and, ultimately, better quality of life. Regular ingestion of baked milk or egg has also been associated with a faster development of tolerance to the fresh form of the food [7]. However, no universally accepted cut-offs for either skin prick test or specific IgE that may predict which children are able to tolerate the baked form of milk or egg exist, so a baked milk or egg challenge remains the gold standard diagnostic tool for baked milk or egg tolerance. Four of our patients were challenged to baked milk (3/18) and baked egg (1/18). The baked egg challenge resulted in the only positive challenge in our cohort. It involved a 22-month old male with prior history of hives on exposure to cooked egg. He reacted after consuming 145 mg of egg protein, with hives on his anterior chest and facial erythema. Following treatment with a single dose of antihistamine all his symptoms resolved.

Infant food challenges differ from those that involve older children or adults. In fact, a AAAAI (American Academy of Allergy, Asthma and Immunology) work group report was produced with the aim to guide clinicians in the conduction of OFCs to peanut in infants [8]. The main concern of parents and clinicians is the possibility of anaphylaxis during an infant OFC, especially because in this age group, the diagnosis of anaphylaxis may present some added diagnostic challenges. Very young children are unable to describe certain symptoms such as pruritus or throat tightness. Certain signs such as irritability and behavioral changes can be difficult to interpret as they may also occur in healthy infants [9]. In young children aged 0–2 years, cow’s milk and egg are the most commonly reported foods implicated in anaphylactic episodes [10], followed by peanuts, sesame, and fish [9,11]. We did not observe any episodes of anaphylaxis in our cohort of patients and the only positive challenge resulted in mild cutaneous symptoms. 

## 5. Conclusions

We suggest that food challenges in infants and young children up to the age of 2 years are generally safe; symptoms appear to be limited to the skin when reactions occur. This includes high-risk infants with prior allergic reactions as well as infants with no known exposure to the food challenged. In carefully selected populations, the large majority of food challenges are likely to be negative, with most infants being sensitized rather than allergic to the food. However, our patient sample is small and further studies will be required to confirm these findings.

## Figures and Tables

**Table 1:** Infant oral food challenges: demographics, atopic history and oral food challenge information.

N	Age (Months)	Gender	Food	SPT Extract (mm)	SPT Fresh (mm)	Specific IgE (kU/L)	Eczema	Allergic Rhinitis	Asthma	Other Food Allergy	Symptoms of Index Reaction	Challenge Result
1	13	M	Baked milk	6	10	1.63	No	No	No	Egg	Hives	Negative
2	19	M	Baked milk	8	10	0.63	Yes	No	No	No	Hives, angioedema, vomiting	Negative
3	8	M	Peanut	3	-	0.1	Yes	No	No	No	No prior ingestion	Negative
4	14	M	Baked milk	10	18	0.2	Yes	No	No	Egg, peanut	Eczema flare	Negative
5	15	M	Soy	20	1	0.26	Yes	No	No	Egg, peanut	No prior ingestion	Negative
6	22	M	Baked egg	0	0	11.1	No	Yes	No	Milk	Hives	Positive
7	13	M	Soy	0	20	0.89	Yes	No	No	Egg, peanut, wheat	Hives, difficulty in breathing	Negative
8	10	M	Peanut	0	0	0.12	Yes	No	No	Egg	No prior ingestion	Negative
9	14	M	Sesame	0	3	0.94	Yes	No	No	Egg, peanut, wheat	Hives, difficulty in breathing	Negative
10	10	M	Peanut	3	0	-	Yes	No	No	Milk	No prior ingestion	Negative
11	10	F	Almond	2	0	0.21	Yes	No	No	Peanut	No prior ingestion	Negative
12	12	M	Almond	3	0	0.34	Yes	No	No	Egg, peanut, wheat	No prior ingestion	Negative
13	15	M	Peanut	4	4	-	Yes	No	No	No	No prior ingestion	Negative
14	12	M	Peanut	2	0	0.55	Yes	No	No	Tree nuts	No prior ingestion	Negative
15	34	M	Hazelnut	2	0	1.34	Yes	No	No	Walnut, pecan	No prior ingestion	Negative
16	25	M	Walnut	3	0	0.1	Yes	No	Yes	Egg, peanut	No prior ingestion	Negative
17	27	M	Cashew	5	4	0.37	Yes	No	Yes	Egg, peanut	No prior ingestion	Negative
18	8	F	Peanut	1	0	0.1	Yes	No	No	No	Eczema flare, itching, irritability	Negative

M: Male, F: Female.
